# Symptomatic Radiation Pneumonitis After Stereotactic Body Radiation Therapy for Multiple Pulmonary Oligometastases or Synchronous Primary Lung Cancer

**DOI:** 10.1016/j.adro.2022.100911

**Published:** 2022-02-05

**Authors:** Noriko Kishi, Yukinori Matsuo, Masahiro Yoneyama, Kazuhito Ueki, Takashi Mizowaki

**Affiliations:** Department of Radiation Oncology and Image-Applied Therapy, Graduate School of Medicine, Kyoto University, Kyoto, Japan

## Abstract

**Purpose:**

Stereotactic body radiation therapy (SBRT) can be easily used for patients with tumors in various organs and is a promising local therapy for eradicating tumors in cancer patients. There is a rising clinical need for increasing knowledge of oligometastases in the treatment of multiple pulmonary tumors. This study aimed to explore the predictive factors for symptomatic radiation pneumonitis (RP) after SBRT for multiple pulmonary oligometastases or synchronous primary lung cancer (SPLC).

**Methods and Materials:**

A total of 38 consecutive patients who had 2 or more pulmonary oligometastases (n = 21) or SPLC (n = 17) and who were treated with SBRT were investigated. Patient characteristics, tumor characteristics, and details of radiation therapy were retrospectively collected from a clinical database. The association between RP of grade 2 or worse (grade 2+ RP) and clinical or dosimetric factors was assessed using logistic regression analyses.

**Results:**

The tumors presented ipsilaterally in 24 patients and bilaterally in 14 patients. During the median follow-up period of 4.9 years, grade 2+ RP, grade 2 RP, and grade 3 RP were observed in 9 patients (23.7%), 7 patients (18.4%), and 2 patients (5.3%), respectively. The mean lung dose (MLD) and the volume of the normal lung receiving ≥5 Gy (lung V_5Gy_) were significantly associated with grade 2+ RP (*P* = .023 and *P* = .012, respectively). The logistic model showed that 20% and 50% of the predicted probability of grade 2+ RP were 6.1 Gy and 9.1 Gy for MLD and 31.6 % and 42.8% for lung V_5Gy_, respectively.

**Conclusion:**

Although further investigation is required to validate the metrics and establish reliable dose constraints, the dose-volume metrics for the normal lung could be predictive of the development of grade 2+ RP after SBRT for multiple pulmonary oligometastases or SPLCs.

## Introduction

The concept of oligometastasis was proposed in 1995. It is a clinical state in which the anatomy and physiology may limit or concentrate metastases to a single or limited number of organs.^1^ The treatment strategy for patients with metastatic tumors is systemic therapy, owing to the spread of microscopic tumor cells. However, local therapy for oligometastatic lesions has recently been explored as a curative approach to eradicating tumors in cancer patients.

Stereotactic body radiation therapy (SBRT) is a local therapy with a precise irradiation technique that delivers high radiation doses to small, targeted tumors while decreasing the irradiated dose to organs at risk surrounding the tumor. It is noninvasive and is performed in an outpatient setting with a 1- or 2-week treatment duration. Therefore, it can be easily used for patients with tumors in various organs, such as the lungs, liver, adrenal glands, kidneys, brain, and bone. A randomized phase 2 trial, SABR-COMET, investigated the effectiveness and toxic effects of SBRT in patients with a controlled primary tumor and fewer than 5 oligometastatic lesions.^2^^,^^3^ The lesional control was increased by 26% in the SBRT group compared with the control group (75 of 100 lesions [75%] vs 28 of 57 lesions [49%]). In addition, overall survival (OS) in the SBRT group significantly improved compared with that in the control group (42.3% vs 17.7% at 5 years; *P* = .006). The most common target lesions in the SBRT group were lung metastases (55 patients [43%]), although grade 5 pulmonary toxic effects were observed in 2 patients.

Pulmonary toxic effects are a significant issue in patients with pulmonary lesions treated with SBRT. Radiation pneumonitis (RP) is the most common pulmonary toxic effect, and there is an association between the irradiated volume and RP.^4^, ^5^, ^6^ A UK consensus recommends the following: (1) lung (normal lungs minus gross tumor volume) V_20Gy_ < 12.5% is optimal (V*_x_*_Gy_ is the volume of the organ at risk receiving ≥*x* Gy), and < 15% is acceptable when patients with more than 1 lung lesion are treated with SBRT; and (2) these lesions should be treated on alternate days and with the same dose and fractionation.^7^, ^8^, ^9^ In clinical practice, SBRT is sometimes delivered in relatively large target volumes using a different dose and fractionation, depending on the timing of systemic therapy, the general condition of the patient, and the tumor locations. In such cases, it is unclear whether SBRT can be applied to 2 or more pulmonary lesions without increasing the incidence of RP and compromising lesion control. Only a few studies have reported the association between the irradiated dose and volume to normal lungs and the risk of developing RP in patients with 2 or more pulmonary lesions.^10^, ^11^

As oligometastases become increasingly recognized in the clinical field, the need for SBRT in the treatment of multiple pulmonary tumors may also increase. This study aimed to explore the predictive factors for symptomatic RP in patients with multiple pulmonary tumors who were treated with SBRT.

## Methods and Materials

This study was performed in accordance with the Declaration of Helsinki (1975, as revised in 2013). The study protocol was approved by the Kyoto University Ethics Committee in 2021. Consent was obtained in the form of opting out. The requirement for written informed consent was waived owing to the study's retrospective nature.

### Patient selection

Data from consecutive patients treated at our institute between January 2003 and December 2020 were used for investigation. The inclusion criteria for patients were as follows: (1) presence of 2 or more pulmonary oligometastases or synchronous primary lung cancer (SPLC)^12^ and (2) having undergone treatment with SBRT in a single course (including multiple lesions treated alternately, sequentially, and concurrently). Patients who had viable lesions other than the target lesions or who terminated the planned treatment early were excluded from the analysis.

### Treatment protocol

The details of our SBRT procedure for lung cancer and oligometastatic pulmonary tumors have been previously reported.^13^ In brief, the patient was immobilized with a stereotactic body frame. The internal target volume was determined based on computed tomography (CT) with a slow-scan technique until June 2009 and was based on 4-dimensional CT thereafter. Tumor motion was assessed using x-ray fluoroscopy. A 5-mm margin was added to the internal target volume to create the planning target volume (PTV). Normal lung was defined as the total lung minus the gross tumor volume. For SBRT, 6-MV x-rays were delivered using a linear accelerator in multiple coplanar and noncoplanar beams or intensity modulated volumetric arc therapy. The prescribed doses for peripherally located lesions were as follows: 48 to 56 Gy in 4 fractions or 60 Gy in 5 fractions to the isocenter, which correspond to 42 to 49 Gy or 52.5 Gy at the PTV periphery, respectively, until March 2014 and 70 Gy in 4 fractions to the isocenter, which corresponds to 50 Gy at the PTV periphery, thereafter.^13^ The prescribed dose for centrally located lesions was 60 Gy in 8 fractions to the isocenter, which corresponds to 52.5 to 54 Gy at the PTV periphery. When multiple PTVs were in different lobes or treated with a different fractionation, separate plans were created for each PTV, with the consideration to avoid beam overlap. When the PTVs were closely located in the same lobe, a single plan with 1 isocenter was created, with the multiple PTVs optimized together. Treatment planning was performed with consideration of previously reported dose constraints.^14^ Dose distributions were calculated with a pencil beam convolution algorithm with heterogeneity correction using the Batho power law method until June 2009, x-ray Voxel-Monte Carlo until April 2018, and collapsed cone convolution thereafter, owing to the upgrade of the treatment planning system.

At each visit during the follow-up, physical examinations and chest radiography or CT were performed every 3 to 6 months up to the 5th year and every 6 to 12 months thereafter. When recurrence was suspected, all patients were assessed using brain magnetic resonance imaging and/or fluorodeoxyglucose-positron emission tomography/CT.

The following data were retrospectively collected from the database: patient characteristics (age, sex, Eastern Cooperative Oncology Group performance status, smoking status, the existence of interstitial pneumonia, prior history of lung surgery or use of systemic therapy and/or radiation therapy, and forced expiratory volume in 1 second), tumor characteristics (primary site, number of lesions, laterality, the existence of a lower lobe lesion, and indication for SBRT [SPLC or oligometastatic disease]), and details of radiation therapy (prescribed dose at the periphery, the timing of irradiation of the multiple PTVs, treatment period, summed PTV volume, mean lung dose [MLD], lung V_5Gy_, and lung V_20Gy_). The dose-volume metrics for the normal lung were evaluated based on the nominal doses, which were calculated from the voxel-wise summation of the multiple treatment plans.

### Statistical analysis

The primary endpoint was symptomatic RP, which refers to radiation pneumonitis of grade 2 or worse (grade 2+ RP). Overall survival, progression-free survival (PFS), the cumulative incidence of local recurrence (LR), and grade 2+ toxic effects other than RP were also investigated. The crude rate of grade 2+ RP and the cumulative incidence rate of grade 2+ RP at 1 year, with death as a competing risk, were both calculated. Overall survival was defined as the period from the initiation of SBRT until the day of death and was censored at the last follow-up. Progression-free survival was defined as the period from the initiation of SBRT to the day of disease progression or death from any cause and was censored at the last follow-up. Local recurrence was defined as tumor recurrence within the PTV and was investigated per lesion. Toxic effects were graded according to the Common Terminology Criteria for Adverse Events, version 5.0.

The median follow-up period was calculated using the reverse Kaplan-Meier method.^15^ Univariate binary logistic regression analysis was applied to determine variables that would predict grade 2+ RP. The correlations between dose-volume metrics for the normal lung were calculated using Pearson correlation analysis. Statistical significance was set at *P* < .05. The logistic model was created based on the determined predictive variables, and the values for 20% and 50% of the predicted probability for grade 2+ RP were calculated. The area under the curve (AUC) was calculated using receiver operating characteristic curves generated from the binary logistic regression model. The OS and PFS were evaluated using the Kaplan-Meier method, and the cumulative incidence of LR was calculated, with death owing to any cause taken as a competing risk. Differences in OS were evaluated using the log-rank test. Statistical analyses were performed using R software, version 4.0.2 (R Foundation for Statistical Computing, Vienna, Austria).

## Results

### Patient characteristics

A total of 38 patients (24 men and 14 women) with a total of 78 lesions were included in the analysis after excluding a patient who did not complete the SBRT course owing to exacerbation of a pre-existing comorbidity (diabetic foot ulcer). The mean age was 72 (range, 30-88) years (Table 1). There were 17 patients (45%) who had a history of lung surgery and 14 (37%) who had prior use of systemic therapy. There was no concurrent use of chemotherapy with SBRT. The indications for SBRT were SPLC in 17 patients and oligometastatic disease in 21 patients; oligometastatic disease was classified as metachronous oligorecurrence in 11 patients, metachronous oligoprogression in 1, induced oligorecurrence in 6, and induced oligoprogression in 3 according to a consensus recommendation.^16^ The primary sites in the oligometastatic cases were lung (22 patients), colorectum (7 patients), head and neck (2 patients), esophagus (2 patients), hepato-biliary-pancreas (2 patients), ovary (2 patients), and kidney (1 patient). The lesions were located in the ipsilateral lung in 24 patients and the bilateral lungs in 14 patients.

**Table 1:** Patient characteristics and univariate analysis

		Radiation pneumonitis		Univariate analysis	
Characteristic	All cases (n = 38)	Grade 0-1(n = 29)	Grade 2+(n = 9)	OR (95% CI)	*P* value
Age, mean (range), y	72 (30-88)	72 (30-86)	73 (56-88)	1.01 (0.94-1.10)	.86
Sex					
Male	24	20	4	0.36 (0.07-1.66)	.19
Female	14	9	5		
ECOG-PS					
1+	18	12	6	2.83 (0.62-15.6)	.19
0	20	17	3		
Smoking history*					
Yes	25	19	6	1.26 (0.23-9.89)	.80
No	10	8	2		
Interstitial pneumonia					
Yes	3	3	0	NA	NA
No	35	26	9		
Prior history of lung surgery					
Yes	17	15	2	0.27 (0.04-1.33)	.14
No	21	14	7		
Prior use of systemic therapy					
Yes	14	10	4	1.52 (0.31-7.07)	.59
No	24	19	5		
Prior use of radiation therapy					
Yes	2	2	0	NA	NA
No	36	27	9		
FEV_1_,† mean (range), L	1.80 (0.93-4.29)	1.87 (0.99-4.29)	1.49 (0.93-2.33)	0.38 (0.04-1.77)	.31
Lesions, n					
Three	2	2	0	NA	NA
Two	36	27	9		
Laterality					
Ipsilateral	24	17	7	2.47 (0.49-18.6)	.31
Bilateral	14	12	2		
Existence of lower lobe lesion					
No	15	13	2	2.84 (0.57-21.3)	.24
Yes	23	16	7		
Indication for SBRT					
SPLC	17	12	5	1.77 (0.39-8.52)	.46
Oligometastatic disease	21	17	4		

*Abbreviation*s*:* CI = confidence interval; ECOG-PS = European Cooperative Oncology Group performance status; FEV_1_ = forced expiratory volume in 1 second; NA = not applicable; OR = odds ratio; SBRT = stereotactic body radiation therapy; SPLC = synchronous primary lung cancer.

⁎ Data on smoking status were unavailable for 3 patients.

† Data on FEV_1_ were unavailable for 11 patients.

The median treatment period was 8 (range, 4-15) days. In 22 patients, all PTVs were irradiated at the same time (concurrently). In 10 patients, 1 PTV was irradiated after the other PTV (sequentially). The remaining 6 patients received irradiation to 1 PTV and the other on alternate days (alternately). The detailed characteristics of the radiation therapy are shown in Table 2.

**Table 2:** Details of radiation therapy and univariate analysis

Variable	All cases(n = 38)	Radiation pneumonitis		Univariate analysis	
		Grade 0-1(n = 29)	Grade 2+(n = 9)	OR(95% CI)	*P* value
Treatment period, mean (range), d	8 (4-15)	8 (4-15)	8 (4-10)	0.99 (0.76-1.28)	.93
Prescribed dose at periphery					
42-50 Gy in 4 fractions	29	22	7	0.90 (0.12-4.86)	.91
Other fractionations	9	7	2		
Timing of irradiation of the multiple PTVs					
Concurrently	22	16	6	1.63 (0.35-8.90)	.54
Sequentially or alternately	16	13	3		
Summed PTV, mean (range), cm^3^	63.6 (16.2-179.0)	61.8 (16.2-179.0)	69.3 (25.9-111.3)	1.00 (0.99-1.02)	.66
MLD, mean (range), Gy	6.0 (2.6-11.2)	5.6 (2.8-10.5)	7.5 (2.6-11.2)	1.59 (1.09-2.50)	.023
Lung V_5Gy_, mean (range), %	30.6 (12.1-54.2)	28.0 (14.7-45.5)	39.1 (12.1-54.2)	1.13 (1.04-1.27)	.012
Lung V_20Gy_, mean (range), %	8.4 (2.0-19.0)	7.6 (2.0-19.0)	11.0 (4.9-18.9)	1.18 (1.00-1.43)	.052

*Abbreviations:* CI = confidence interval; lung V*_x_*_Gy_ = the volume of the normal lung receiving ≥*x* Gy; MLD = mean lung dose; OR = odds ratio; PTV = planning target volume.

### Grade 2+ radiation pneumonitis and other toxic effects

**Fig. 1:**
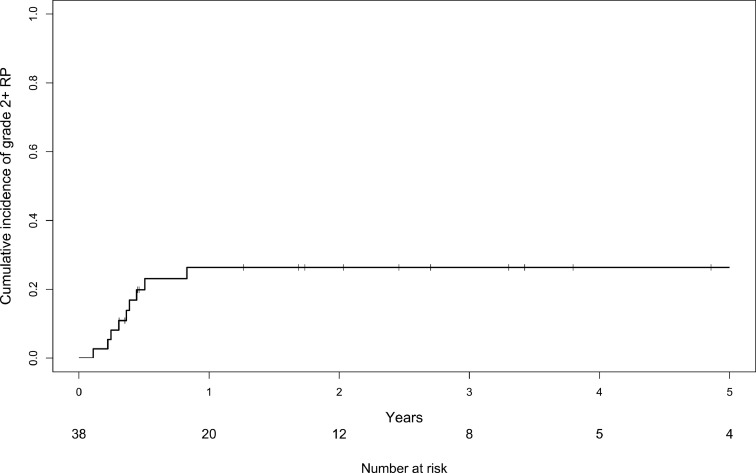
The cumulative incidence of radiation pneumonitis of grade 2 or worse (grade 2+ RP) with death as a competing risk.

Grade 2+ RP was observed in 9 patients (crude rate, 23.7%), grade 2 RP in 7 patients (18.4%), and grade 3 RP in 2 patients (5.3%). Grade 2+ RP occurred within 10.0 months after SBRT (Fig 1), and only 2 patients died during this period. The cumulative incidence rate of grade 2+ RP at 1 year was 26.4%. Six patients received home oxygen therapy 2.8 years after SBRT (range, 0.3-10.1 years). Among them, 2 patients developed grade 3 RP.

**Fig. 2:**
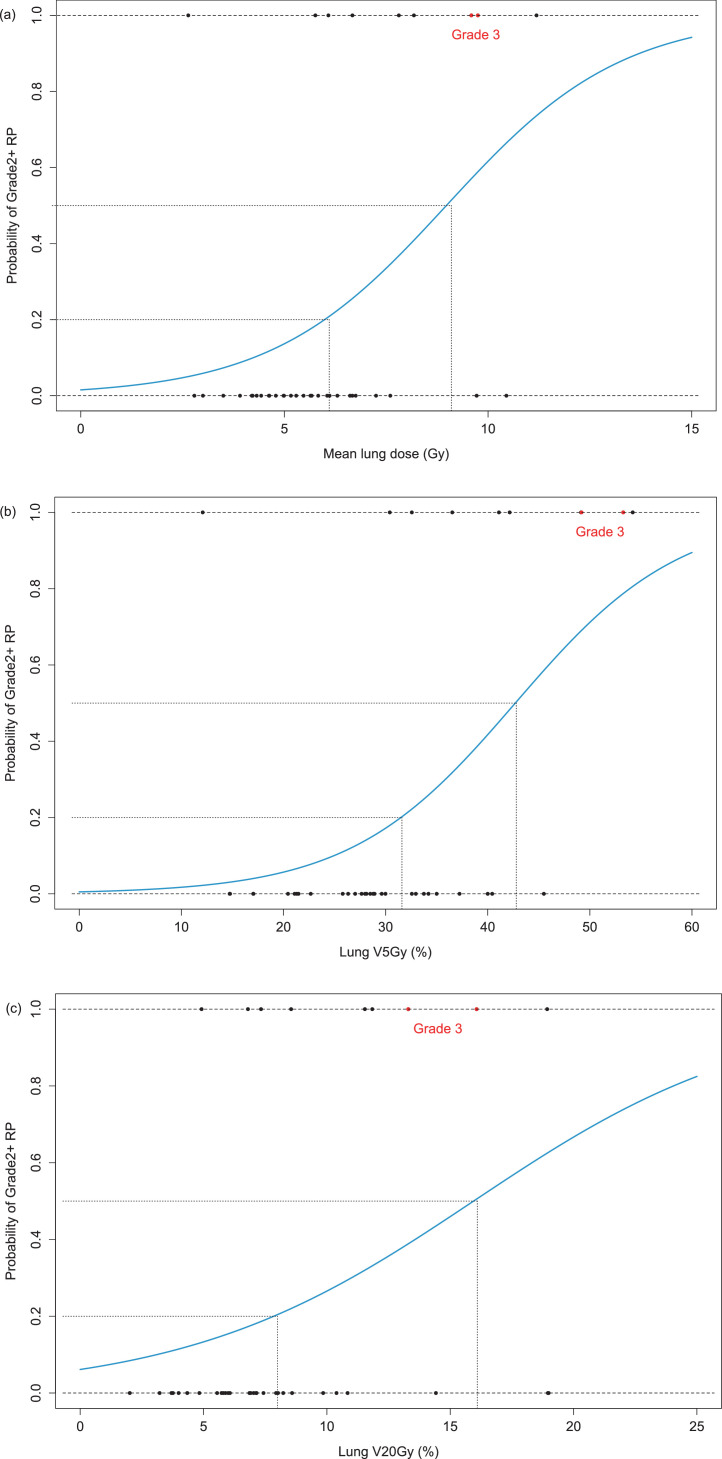
Logistic regression curves of the predicted probability of radiation pneumonitis of grade 2 or worse (grade 2+ RP) based on (a) mean lung dose (MLD), (b) lung V_5Gy_, and (c) lung V_20Gy_. The black circles indicate each patient with grade 2 RP, and the red circles indicate each patient with grade 3 RP. The dotted lines indicate that the 20% and 50% predicted probabilities for grade 2+ RP are 6.1 Gy and 9.1 Gy for MLD, 31.6% and 42.8% for lung V_5Gy_, and 8.0% and 16.1% for lung V_20Gy_, respectively. *Abbreviations:* lung V*_x_*_Gy_ = the volume of the normal lung receiving ≥*x* Gy.

**Fig. 3:**
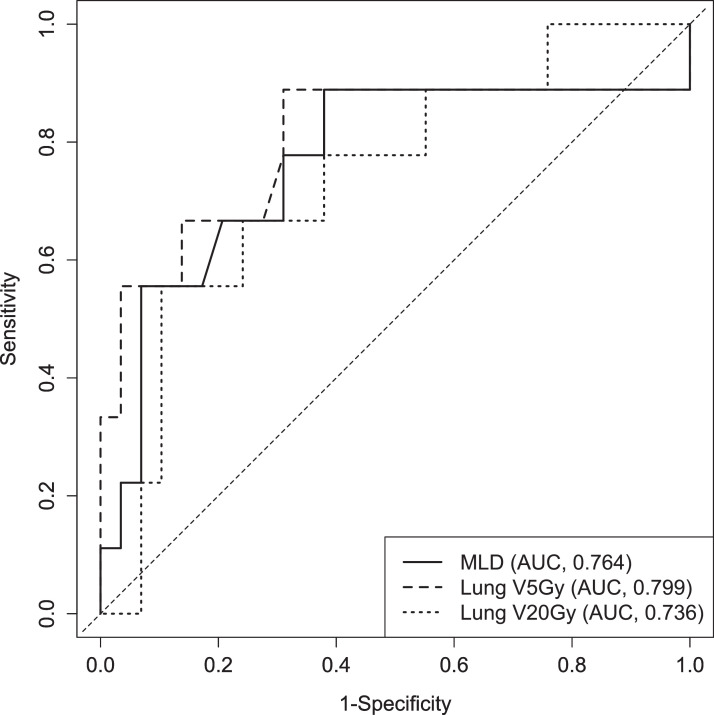
The receiver operating characteristic curves of the mean lung dose (solid line), lung V_5Gy_ (thick dotted line), and lung V_20Gy_ (dotted line). *Abbreviations:* AUC = area under the curve; lung V*_x_*_Gy_ = the volume of the normal lung receiving ≥*x* Gy; MLD = mean lung dose.

Univariate analysis showed that there were no significant differences between patients who developed grade 0 to 1 RP and grade 2+ RP in terms of age, sex, Eastern Cooperative Oncology Group performance status, smoking history, interstitial pneumonia, prior history of lung surgery or use of systemic therapy and/or radiation therapy, forced expiratory volume in 1 second, indication for SBRT, number of lesions, and ipsilateral or bilateral location. As for the dose-volume metrics, MLD and lung V_5Gy_ were significantly associated, and lung V_20Gy_ was marginally associated with grade 2+ RP (*P* = .023, *P* = .012, and *P* = .052, respectively; Table 2). The logistic models showed that the values for 20% and 50% of the predicted probability of grade 2+ RP were 6.1 Gy and 9.1 Gy for MLD (Fig 2a), 31.6 % and 42.8% for lung V_5Gy_ (Fig 2b), and 8.0% and 16.1% for lung V_20Gy_ (Fig 2c), respectively. The AUCs for MLD, lung V_5Gy_, and lung V_20Gy_ were 0.764 (95% confidence interval [CI], 0.546-0.983), 0.799 (0.580-1.000), and 0.736 (0.545-0.927), respectively. There was no significant difference in the AUCs between the 3 metrics (MLD vs lung V_5Gy_, *P* = .33; lung V_5Gy_ vs lung V_20Gy_, *P* = .43) (Fig 3). The 3 dose-volume metrics for the normal lung were highly correlated with each other, with correlation coefficients (*r*) of 0.90 (95% CI, 0.82-0.95; *P* < .001), 0.88 (95% CI, 0.78-0.94; *P* < .001), and 0.70 (95% CI, 0.49-0.83; *P* < .001) between the MLD and lung V_5Gy_, MLD and lung V_20Gy_, and lung V_5Gy_ and lung V_20Gy_, respectively.

Grade 2+ toxic effects other than RP were observed in 6 patients: grade 2 rib fracture in 3 patients, grade 2 dermatitis in 1 patient, grade 3 pleural effusion in 1 patient, and grade 3 lung infection in 1 patient.

### Overall survival, progression-free survival, and cumulative incidence of local recurrence

**Fig. 4:**
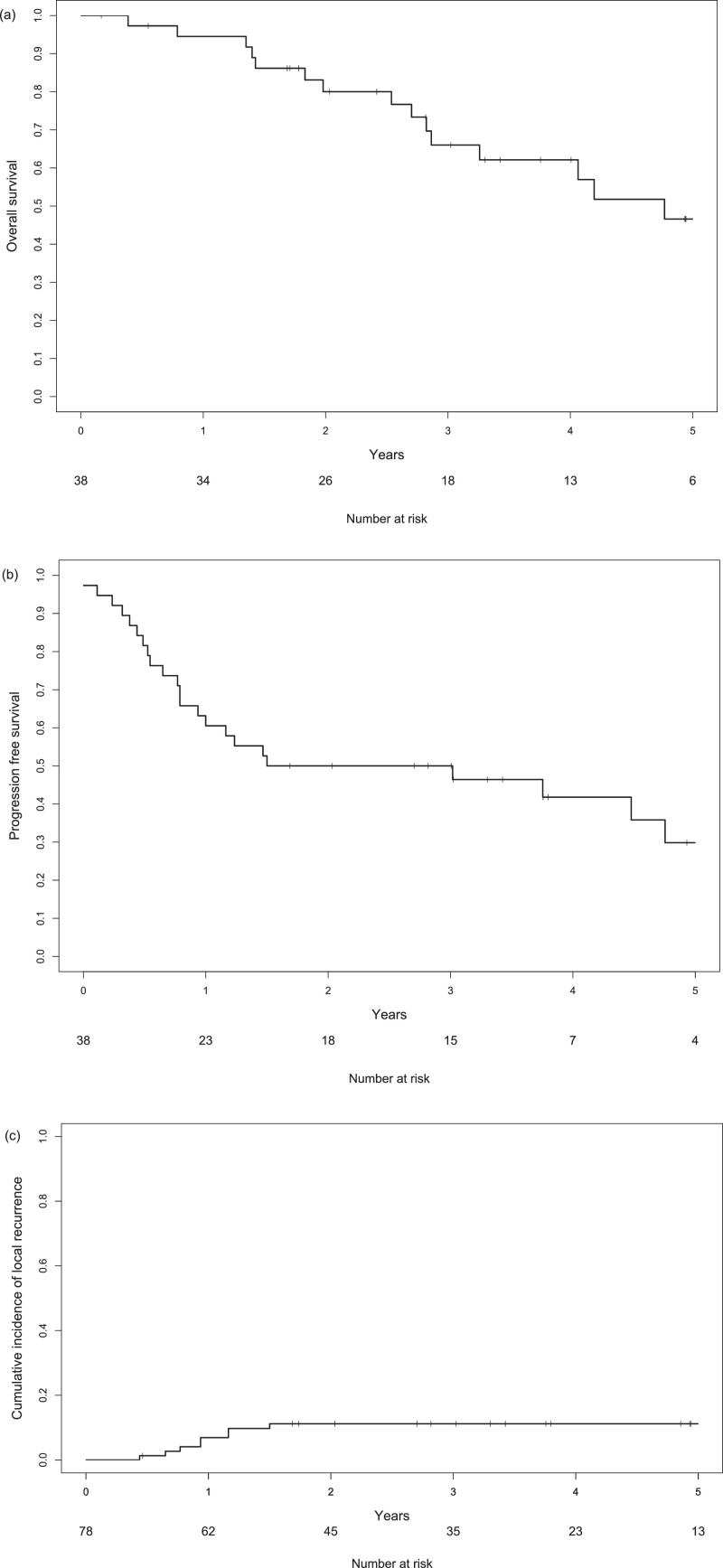
Kaplan-Meier curves of (a) overall survival, (b) progression-free survival, and (c) the cumulative incidence of local recurrence.

The median survival time was 4.8 years (95% CI, 2.9 years to not reached) during the potential follow-up period of 4.9 (95% CI, 3.3-8.0) years. The 3-year OS and PFS were 66.0% (95% CI, 51.3%-84.9%) and 50.0% (95% CI, 36.4%-68.7%), respectively (Fig 4). There was no significant difference in OS between patients with grade 0 to 1 RP and grade 2+ RP (3-year OS, 66.6% vs 66.7%, respectively; *P* = .30).

LR was observed in 7 patients and in 9 of 78 total treated lesions. The cumulative incidence of LR after 3 years was 11.1% (95% CI, 5.8%-21.5%).

## Discussion

The role of local therapy in oligometastases has become increasingly important in recent years. SBRT is a promising technique for the management of oligometastases and is often administered to patients with multiple pulmonary tumors. However, a major concern in performing SBRT for multiple pulmonary tumors is the increased risk of RP. In this study, we demonstrated that the dose-volume metrics for the normal lung could be predictive of grade 2+ RP in patients with multiple pulmonary oligometastases or SPLC who were treated with SBRT in a single course.

For single pulmonary lesions, the incidence of grade 2+ RP is less than 10%.^4^^,^^5^ Various clinical predictive factors have been reported, including previous anatomic lung resection,^17^ pretreatment pulmonary interstitial change,^18^ and dose-volume metrics. The known dose-volume metrics are the PTV, lung V_25Gy_,^19^ lung V_20Gy_,^4^ and MLD.^5^ In this study, the MLD and lung V_5Gy_ were found to be significantly associated with grade 2+ RP and lung V_20Gy_ was marginally associated, whereas prior lung surgery, the existence of interstitial pneumonia, and the PTV were not associated. Previous reports on multiple pulmonary tumors have included patients with various numbers of treatment courses and lesions. Therefore, the reported incidence of RP varies widely. Owen et al^20^ investigated 63 patients with 128 lesions (including 21 patients with multiple pulmonary lesions) and reported that grade 3+ RP occurred in 2 patients (3.2%). Moding et al^21^ investigated 86 patients with 209 lesions (including 46 patients with multiple treatment courses) and reported that the 4-year incidence of grade 2+ RP was 7.9%. In addition, Shintani et al^22^ reported that in 18 patients (including 15 patients with SPLC treated with SBRT), the incidence of grade 2+ RP was 16% and that of grade 3+ RP was 11%. In this study, we confined the enrolled patients to those who had multiple pulmonary tumors treated with SBRT in a single course, and we found that the incidence of grade 2+ RP was higher than 20%.

When performing SBRT for multiple pulmonary tumors, the dose to the normal lungs tends to be higher than that of SBRT for single pulmonary tumors. Considering the predictive dose-volume metrics for single pulmonary tumors, the incidence of RP in patients with multiple pulmonary tumors could be increased. However, only a few reports have focused on the association between dose-volume metrics and RP in this particular cohort. Muller et al^10^ reported that in 44 patients with 100 lesions treated with 2 courses of SBRT, the incidence of grade 2+ RP and grade 3+ RP were 13.6% and 4.5%, respectively. The dose-volume metrics in the report by Muller et al (PTV of 35.7 cm^3^ for the first course and 21.2 cm^3^ for the second course; MLD, 6.3 Gy) were similar to those in the present study (summed PTV volume, 63.6 cm^3^; MLD, 6.0 Gy). Our results suggest that single-course irradiation for multiple pulmonary tumors could increase the risk of developing RP, although the incidence of grade 3+ RP was similar between the studies (4.5% vs 5.3%). An MLD > 9 Gy, V_5Gy_ > 40%, or V_20Gy_ > 16% could increase the risk of developing grade 2+ RP to as high as approximately 50%; if the dose-volume metrics for the normal lung can be reduced to MLD < 6 Gy, V_5Gy_ < 30%, and V_20Gy_ < 8%, the risk of developing grade 2+ RP would be decreased. The incidence of grade 2+ RP was estimated to be < 20% in our study. Considering these results, in cases with a low irradiated lung dose, SBRT for multiple pulmonary tumors in a single course might be performed with acceptable toxic effects.

The local control (LC) rate of SBRT for pulmonary lesions is high, although slightly different rates are shown depending on the histology: the LC rate at 3 years was >85% in solitary primary lung cancer,^14^ and the LC rate at 2 to 3 years was 59% to 80% in oligometastatic pulmonary tumors.^23^, ^24^, ^25^, ^26^, ^27^ A biological effective dose with an α/β ratio of 10 Gy (BED_10_) < 100 Gy to the target volume and pulmonary metastasis that originates from tumors less responsive to radiation therapy, such as colorectal cancer, are associated with a poor LC rate.^24^^,^^25^^,^^28^^,^^29^ As for OS, previous reports on oligometastatic pulmonary tumors showed widely ranging OS rates of 50% to 70% at 2 to 3 years.^23^^,^^24^^,^^26^ In this study, 6 patients (15%) were treated with 8 fractions (BED_10_ < 100 Gy) owing to the central location, and 8 patients (21%) with pulmonary metastasis that originated from tumors less responsive to radiation therapy (colorectal cancer and renal cancer) were included. Although a direct comparison between the studies is difficult owing to the different patient backgrounds and treatment, the incidence of LR in this study was relatively low compared with these previous reports, and the OS was comparable. The treatment indication for SBRT for multiple pulmonary lesions in our institute could be considered appropriate in terms of OS.

This study had several limitations. First, this was a retrospective study that involved a small sample size. Second, treatment plans with different dose calculation algorithms were included in this study, which could cause an error of ≤1% in the mean dose of the hemi-lung and of ≤1% in the PTV.^30^^,^^31^ Third, although it is known that the irradiation dose to the heart is significantly associated with noncancer death, dose-volume metrics related to the heart were not investigated.^32^ Further investigation is needed to determine appropriate candidates for SBRT among patients with multiple pulmonary tumors, while considering a balance between efficacy and toxic effects.

The clinical need for SBRT as treatment for multiple pulmonary oligometastases is increasing. When planning a prospective study to establish an aggressive treatment strategy for multiple pulmonary oligometastases, the dose constraints for normal lungs, as well as patient selection criteria, should be carefully considered.

## Conclusion

Although further investigation is needed to validate the metrics and establish reliable dose constraints, the dose-volume metrics for the normal lung could be predictive of development of grade 2+ RP after SBRT for multiple pulmonary oligometastases or SPLCs.
